# Liquid–liquid extraction of uranium(VI) in the system with a membrane contactor

**DOI:** 10.1007/s10967-013-2796-0

**Published:** 2013-10-25

**Authors:** Paweł Biełuszka, Grażyna Zakrzewska, Ewelina Chajduk, Jakub Dudek

**Affiliations:** Institute of Nuclear Chemistry and Technology, Dorodna 16, 03-195 Warsaw, Poland

**Keywords:** Uranium, Membrane contactor, Extraction, D2EHPA

## Abstract

Raising role of the nuclear power industry, including governmental plans for the construction of first nuclear power plant in Poland, creates increasing demand for the uranium-based nuclear fuels. The project implemented by Institute of Nuclear Chemistry and Technology concerns the development of effective methods for uranium extraction from low-grade ores and phosphorites for production of yellow cake—U_3_O_8_. The Liqui-Cel^®^ Extra-Flow 2.5 × 8 Membrane Contactor produced by CELGARD LLC (Charlotte, NC) company is the main component of the installation for liquid–liquid extraction applied for processing of post leaching liquors. In the process of membrane extraction the uranyl ions from aqueous phase are transported through the membrane into organic phase. The flow of two phases in the system was arranged in co-current mode. The very important element of the work was a selection of extracting agents appropriate for the membrane process. After preliminary experiments comprising tests of membrane resistivity and determination of extraction efficiency, di(2-ethylhexyl)phosphoric acid was found to be most favourable. An important aspect of the work was the adjustment of hydrodynamic conditions in the capillary module. To avoid the membrane wettability by organic solvent and mixing two phases equal pressure drops along the membrane module to minimize the transmembrane pressure, were assumed. Determination of pressure drop along the module was conducted using Bernoulli equation. The integrated process of extraction/re-extraction conducted in continuous mode with application of two contactors was designed.

## Introduction

Uranium is a relatively abundant chemical element with the highest atomic number of all naturally-occurring elements. It is located in the IIIB group, in the row of actinides of a periodic table. In nature uranium occurs as three radioactive isotopes, namely U-238 (99.28 %), U-235 (0.72 %), and U-234 (0.0055 %). The U-235 isotope is fissile; its nucleus can be split by thermal neutrons releasing much energy and producing more neutrons, which under the right circumstances can lead to a self-sustaining chain reaction utilised in nuclear reactors.

The most important minerals of uranium are: uraninite UO_2_ + UO_3_, carnotite K_2_(UO_2_)_2_(VO_4_)_2_·2H_2_O, brannerite (UTi_2_O_6_), coffinite (USiO_4_·*n*H_2_O), and uranophane (H_3_O)Ca[UO_2_][SiO_4_]_2_ [1, 2]. Uranium is also found in phosphate rocks, lignite, monazite sands and can be recovered commercially from these sources [1]. An important step for obtaining uranium oxide from ores is purification of uranium after leaching and concentration with use of known physical and chemical methods. Solvent extraction and ion exchange are well developed and commercially used processes of separation of uranium from post-leaching solutions. Treatment involves removal of associated metals, such as molybdenum, vanadium, iron, arsenic, zinc, copper, nickel and rare earth elements. At the same time leads to concentration of the solution, from which precipitation of the end-product: diuranate (sodium or ammonium) or triuranium octoxide, depending on the used reagents, is performed. The leaching of the ore is usually carried out either by sulphuric acid or sodium carbonate [1, 3]. For purification and concentration of the solution the most commonly used processes are: liquid–liquid extraction, ion exchange, integrated processes, such as ion exchange/liquid–liquid extraction.

**Fig. 1:**
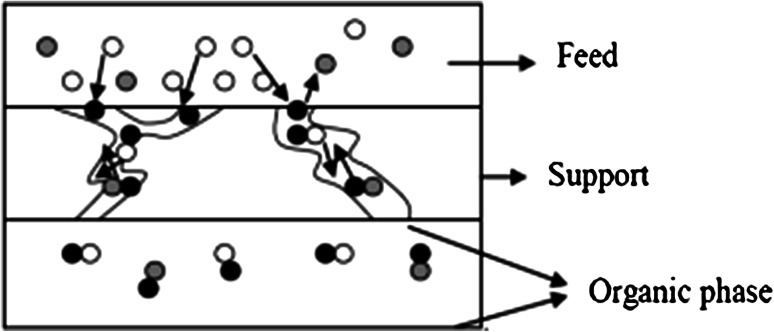
The extraction process using porous hollow fibers to separate the aqueous feed solution physically from the organic solution (*white*, *gray*, and *black dots* represent the impurity ion, the counter ion, and the carrier, respectively)

The new approach for the liquid–liquid extraction of uranium will involve the membrane enclosed in a small volume of the device—the membrane module. The term “membrane contactor” is used to identify membrane systems that are employed to “keep in contact” two phases. On the contrary of the more “traditional” idea of membranes as media for performing separations thanks to their selectivity, membrane contactors do not offer any selectivity for a particular species with respect to another, but simply act as a barrier between the phases involved, by allowing their contact in correspondence of well-defined interfacial area. The two phases are separated by the membrane and species are transferred from one phase to the other by only diffusion. Extraction with the use of membrane contactors has many advantages above conventional methods of the extraction of uranium, like no fluid/fluid dispersion, no emulsion formation, no flooding at high flow rates, low solvent holdup, known and constant interfacial area, easy upscaling, etc. One of the biggest drawbacks of membrane extraction may be expected from the formation of concentration polarization and fouling [4]. There is also the risk of wetting the membranes during long-term operation of the module resulting in mixing of the two phases. For the proper operation of membrane contactors it is important to maintain appropriate hydrodynamic conditions for flow of solutions over the membrane surface in order to eliminate such a danger [5, 6]. In a hollow fiber membrane contactor, the organic phase is immobilized in a porous polymeric support, like a polypropylene hollow fiber, preventing emulsification of the organic phase in the feed solution. This is shown in Fig. 1.

During the extraction in the membrane contactor, ions are received by the organic phase from the feed (aqueous phase) until thermodynamic equilibrium is reached. At equilibrium, the distribution coefficient, *D*_c_ is calculated by:

1 $$ D_{\text{c}} = \frac{{C_{\text{o}} }}{{C_{\text{w}} }} $$

where *C*_o_ is concentration of the component in organic phase, *C*_w_ is concentration of the component in water phase.

Most of the published studies on extraction of metals using membrane contactors were carried out with use of the Liqui-Cel^®^ Extra-Flow modules produced by CELGARD company. Prepasawat et al. [7] studied the simultaneous extraction and back-extraction of As(III) and As(V) from sulphate media using Cynaex-923 extractant in toluene as a metal carrier and water as a stripping phase. Also in these studies, the commercial Liqui-Cel^®^ Extra-Flow modules were used [8]. These modules contain microporous hollow fibers made of a polypropylene (PP). The PP polymer exhibits very stable thermal properties and is resistant to a wide range of organic compounds. The Liqui-Cel ^®^ Extra-Flow modules have a central shield in the shape of the baffle, which on one hand increases the efficiency of the process, but on the other hand results in a velocity component in the perpendicular direction to the membrane surface [9]. St John et al. studied the potential of the D2EHPA/PVC-membrane for the separation of U(VI) from its acidic sulfate solutions [10].

The membranes for membrane contactors are usually microporous and symmetric, and can be both hydrophobic and hydrophilic [11].

## Experimental

### Experimental apparatus

**Fig. 2:**
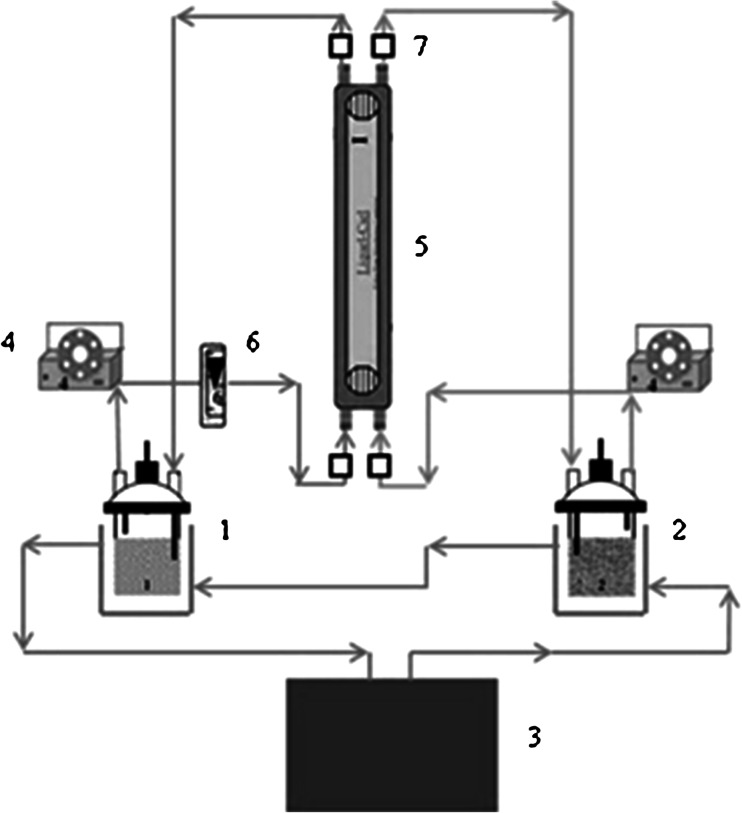
The scheme of the installation for extraction of uranium (*1*) aqueous feed phase; (*2*) organic phase; (*3*) thermostat; (*4*) micropump; (*5*) hollow fiber contactor; (*6*) flow meter; (*7*) temperature sensor

In the present experiments the small, laboratory installation for extraction of uranium equipped with the membrane contactor Liqui-Cel^®^ Extra-Flow 2.5 × 8 produced by CELGARD, was used (Fig. 2). The membrane contactor was connected to two circuits: with aqueous and organic phases. Two micropumps (4) provided fluids from two thermostated liquid reservoirs (1, 2) to the membrane module (5). The system of measuring devices (6, 7), which controlled the parameters of the process (see Fig. 2) was fitted to the unit.

### Extraction experiments

**Fig. 3:**
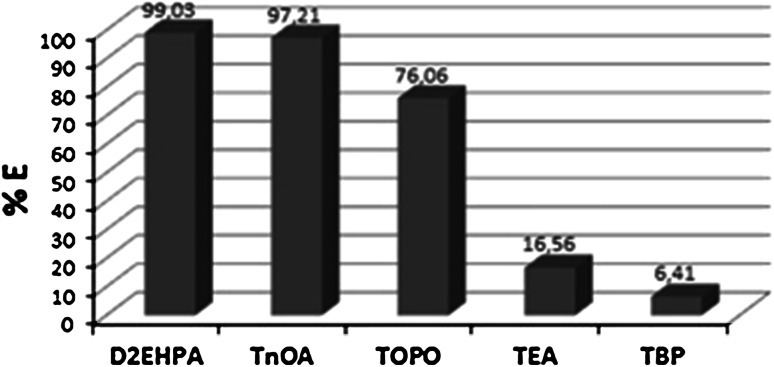
Extraction efficiency of uranium from model solutions of uranyl nitrate (UO_2_(NO_3_)_2_·6H_2_O) in 5 % H_2_SO_4_

A very important part of the work was a selection of uranium extracting agents appropriate for the membrane process. In the laboratory experiments the partition coefficients for uranium extraction by different extractants, like e.g.: tributylphosphate (TBP), triethylamine (TEA), di(2-ethylhexyl)phosphoric acid (D2EHPA), tri-*n*-octylamine (TnOA) and trioctylphosphine oxide were determined. TnOA and D2EHPA were found to be most favourable extractants for uranium in this study; TBP and TEA in the environment of 5 % H_2_SO_4_ were found the weakest extracting agents tested. The classification of the extractants was showed below (see Fig. 3).

After preliminary experiments comprising not only determination of extraction efficiency of selected reagents, but also tests of membrane resistivity, D2EHPA was found to be most favourable for the membrane extraction process. The experiments were carried out with the model solutions of uranyl nitrate in 5 % H_2_SO_4_ using 0.2 M D2EHPA, which was diluted in kerosene or toluene. During the membrane extraction the uranyl ions from aqueous phase were transported through the membrane into organic phase. The flow of two phases was arranged in co-current mode. The organic phase flowed inside thin capillary tubes made of polypropylene, and the aqueous phase washed the capillaries from the outside. The flow of aqueous (feed phase) and organic phase was generated by two micropumps of small yield below 200 L/h. The volume of two phases circulating in the system was 400 mL. The temperature of aqueous and organic phases was controlled by a thermostat and adjusted at 25 °C. Electronic LCD display coupled with PT-100 temperature sensors showed temperature at the inlet and at the outlet of the membrane module. The characteristics of the membrane applied in experiments are shown in Table 1.

**Table 1:** Characteristics of Celgard X50-215 microporous hollow fiber membrane

Material	Polypropylene X-50
Membrane geometry	Capillary
Wall thickness (nominal)	40 μm
Internal diameter (nominal)	220 μm
Outer diameter (nominal)	300 μm
Effective pore size	0.04 μm
Porosity	40 %
Burst strength	400 PSI (15.5 kg/cm^2^)
Total membrane surface area (internal)	1.9 m^2^
Total membrane surface area (outer)	2.6 m^2^
Number of capillaries	11,000

### Selection of process conditions

The crucial element of the work was the appropriate regulation of hydrodynamic conditions in the membrane contactor. This procedure was designed to avoid the membrane wettability by organic solvent and mixing of two phases. Preliminary experiments showed that without proper adjustment of liquid flows in the module the risk of membrane wetting and emulsion formation is potentially high. Spaces, in which two phases—organic and aqueous flow through the module are not symmetric. The liquid flow arrangement inside the capillaries and on the shell side of the module is completely different. When flows of two phases were adjusted at the same level, as usually is done in membrane separation systems, the breakthrough of the membrane occurred and the organic liquid penetrated the membrane pores passing to the aqueous phase. For this reason equal pressure drops along the membrane module to minimize the transmembrane pressure, were assumed. If the pressure difference between the shell and inner side of the fiber wall (transmembrane pressure) exceeds the critical pressure, the organic phase will be pushed out of the most susceptible pores of the support. The system will be unstable and the aqueous feed solution will get contaminated with the organic liquid [11]. Determination of pressure drop along the module was done using Bernoulli equation:

2 $$ \frac{{p_{1} }}{\gamma } + z_{1} + \frac{{u_{1}^{2} }}{{2g\alpha_{1} }} = \frac{{p_{2} }}{\gamma } + z_{2} + \frac{{u_{2}^{2} }}{{2g\alpha_{2} }} + Z_{1,2} $$

where *p*_1_ is pressure at the inlet, *p*_2_ at the outlet of the module (Bar), *α*_1_ and *α*_2_ are Coriolis coefficients (for laminar flow they are equal 0.5 and for turbulent flow are equal 1), *z*_1_ is level of the inlet *z*_2_ of the outlet (m), *γ* is specific weight of the liquid, (kg/m^2^ s^2^). *Z*_1,2_ is resistance to fluid flow along the module:

3 $$ Z_{1,2} = \lambda \frac{{Lu^{2} }}{D2g} $$

where *g* is gravitational acceleration (m/s^2^), *λ* is dimensionless drag coefficient, which is a function of Reynolds number and roughness of the pipe:

4 $$ \lambda = \frac{64}{\text{Re}} $$

5 $$ \text{Re} = \frac{uD}{\nu } $$

where *ν*-kinematic viscosity (m^2^/s), which is expressed as:

6 $$ \nu = \frac{\mu }{\rho } $$

The equations describing pressure drops are as follows:

7 $$ \Updelta p_{1} = \frac{{L(\rho_{1} gd_{{{\text{h}}1}}^{2} + 32\mu_{1} u_{1} )}}{{d_{{{\text{h}}1}}^{2} }} $$

8 $$ \Updelta p_{2} = \frac{{L(\rho_{2} gd_{{{\text{h}}2}}^{2} + 32\mu_{2} u_{2} )}}{{d_{{{\text{h}}2}}^{2} }} $$

Assuming equality of pressure drops along the membrane in organic and water phases:

9 $$ \Updelta p_{1} = \Updelta p_{2} $$

After transformation:

10 $$ u_{2} = \frac{{d_{{{\text{h}}2}}^{2} \mu_{1} }}{{d_{{{\text{h}}1}}^{2} \mu_{2} }}u_{1} + \frac{{(\rho_{1} - \rho_{2} )gd_{{{\text{h}}2}}^{2} }}{{32\mu_{2} }} $$

where *μ*_1_ is dynamic viscosity (physical) of the aqueous phase, *μ*_2_ viscosity of organic phase at temperature 20 °C (kg/m s), *ρ*_1_ is density of the aqueous phase, *ρ*_2_ density of organic phase at 20 °C (kg/m^3^), *u*_1_ is linear flow rate at the inlet and *u*_2_ at the outlet of the fluid (m/s), *L* is the length of the pipe (m), *d*_h1_ is the hydraulic diameter of the part of the module outside of the capillaries (m) and it is expressed as:

11 $$ d_{{{\text{h}}1}} = \frac{{D^{2} - d^{2} - nd_{\text{o}}^{2} }}{{D + nd_{\text{o}} + d}} $$

where *D* is the diameter of the membrane module (m), *d*_o_ is the outer diameter of the capillary (m), *d* is the outer diameter of the empty space in the module (m), *n* is number of capillaries *d*_h2_ is the hydraulic diameter of the part of the module inside of the capillaries:

12 $$ d_{{{\text{h}}2}} = d_{\text{i}} $$

where *d*_i_ is the inner diameter of the capillary (m). After numerous transformations a linear relationship was obtained (see Eq. 13):

13 $$ u_{2} = Au_{1} + B $$

where *A* and *B* are the coefficients in the equation. On the basis of the above relationship the conclusion was formulated that velocity of aqueous and organic phases should remain in close relationships expressed by linear function. The values of the average volumetric flow rates were set with use of the above relationship. For the aqueous phase (feed) the flow rate was equal 98.11 L/h and for organic phase was 5.95 L/h.

## Results and discussion

### Experiments with model solutions

#### Extraction

**Fig. 4:**
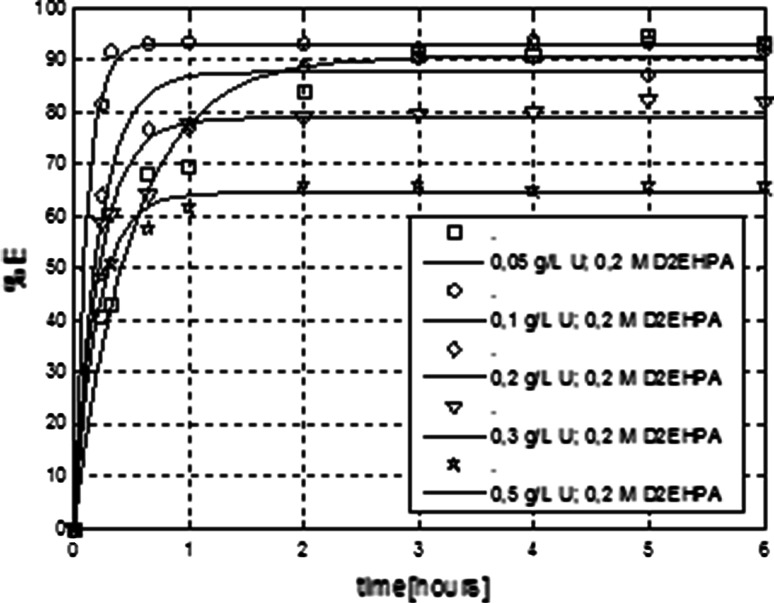
Membrane extraction of uranium using D2EHPA as extractant in toluene, aqueous phase flows inside the capillaries of the membrane contactor

The model solutions of uranyl nitrate UO_2_(NO_3_)_2_·H_2_O were used in the membrane extraction experiments. Chemical analysis was performed applying the method of ICP-MS (Inductively Coupled Plasma Mass Spectrometry). The experimental results were summarized in the kinetic graphs of the extraction efficiency, expressed as a percentage of uranium extracted in time of experiment: %*E* = *f*(*t*). Graphs were approximated according to least squares method:

14 $$ \sum\limits_{i = 1}^{N} {(f(x) - y_{i} )^{2} } $$

where:

15 $$ f(x) = W(1 - \exp ( - t_{i} /T)) $$

and where *W* and *T* are approximation coefficients. The extraction efficiency was calculated by the formula:

16 $$ \% E = \frac{{100D_{\text{c}} }}{{D_{\text{c}} + \frac{{V_{\text{w}} }}{{V_{\text{o}} }}}} $$

where *D*_c_ is a partition coefficient (see Eq. 2), *V*_w_ and *V*_o_ are the volumes of the aqueous and organic phases. The membrane extraction efficiency dependence on time and concentration of uranium (U) in the feed solution is shown in Fig. 4.

**Fig. 5:**
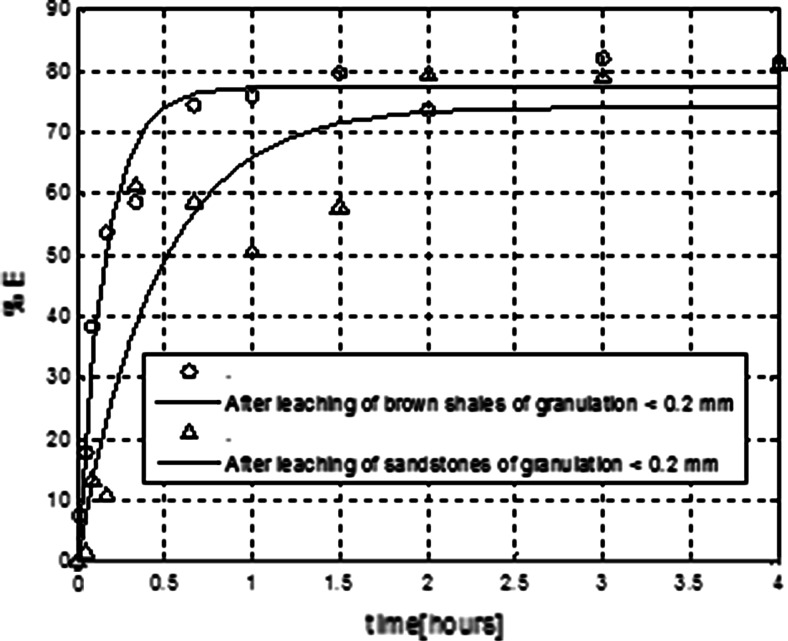
Comparison of membrane extraction of uranium performed with real solutions after acid leaching of brown shales and sandstones

From the results of experiments, it is evident that the kinetics of membrane extraction process using D2EHPA in toluene at different concentrations of uranium is similar. The fastest extraction occured with solutions containing low concentrations of uranium, for example 0.1 g/L (Fig. 4). For this concentration extraction efficiency reached a constant value after less than 1 h. Kinetics of uranium extraction for concentration of 0.05 g/L was different from the other (Fig. 5), however after 2.5 h for all initial concentrations of uranium the equilibrium was established.

#### Extraction/re-extraction

Experiments on membrane re-extraction process were conducted with the same installation (see Fig. 2). In order to carry out the re-extraction experiment, the aqueous phase (feed phase) after membrane extraction was removed from the apparatus. In the place of the removed feed phase the solution of 1 M Na_2_CO_3_ or (NH_4_)_2_CO_3_ was introduced (stripping phase). The volume of stripping phase was the same like feed phase and organic phase (see description of measuring apparatus and extraction experiments). The results of the experiments are shown in Table 2.

**Table 2:** The results of the experiments on extraction/re-extraction process

*C*_a_ (g/L)	*C*_b_ (g/L)	*D*	%*E*	*C*_c_ (g/L)	%reE	%*R*
Stripping phase—(NH_4_)_2_CO_3_						
0.169	0.154	10.51	91.12	0.132	85.71	78.11
0.253	0.236	13.81	93.28	0.198	83.90	78.26
0.485	0.450	12.87	92.78	0.450	100.0	92.78
Stripping phase−Na_2_CO_3_						
0.191	0.177	12.72	92.70	0.145	81.92	75.92
0.263	0.250	19.33	95.06	0.171	68.40	65.02
0.452	0.427	17.15	93.43	0.427	100.0	94.50

In the Table: *C*_a_-concentration of uranium in the feed phase after dilution in the installation, *C*_b_ concentration of uranium in the organic phase after extraction experiment, *C*_c_ concentration of uranium in the stripping phase after re-extraction experiment. %reE percent of uranium re-extraction, was calculated by:

17 $$ \% {\text{reE}} = C_{\text{c}} /C_{\text{b}} \cdot 100\;\% $$

%R percent of recovery of uranium in extraction/re-extraction process was determined by:

18 $$ \% R = C_{\text{c}} /C_{\text{a}} \cdot 100\;\% $$

A comparison of extraction/re-extraction experiments was summarized in Table 2, where initial feed concentration—*C*_a_ had a similar values in re-extraction experiments with (NH_4_)_2_CO_3_ and Na_2_CO_3_. Higher values of %reE and %R were noticed for the re-extraction with (NH_4_)_2_CO_3_ (see Table 2). The highest values of %reE and %R were observed for the higher uranium concentration in the feed solutions—*C*_a_ and in this case %R had higher value for the re-extraction with Na_2_CO_3_ than for (NH_4_)_2_CO_3_.

### Experiments with real post-leaching solutions

#### Extraction

The extraction with real solutions were conducted using the results of the experiments for model solutions. The real solutions were obtained after leaching uranium ores from the Polish samples: (1) sandstones of the Lower and Middle Triassic from Berybaltic Syneclise and (2) black and brown shale from Podlasie Depression. The solutions resulted from treatment of the ores with solutions of 1.88 M sulfuric acid and alkaline solutions of 2 M NaOH/Na_2_CO_3_. The process of acid leaching was conducted by using KMnO_4_ as an oxidant at temperature of 60 °C in 1 h. For the alkaline leaching H_2_O_2_ as an oxidant at temperature of 60 °C was applied and time of leaching was 0.5, 1 and 2 h. In both cases, granulation of ore samples was less than 0.2 mm.

**Fig. 6:**
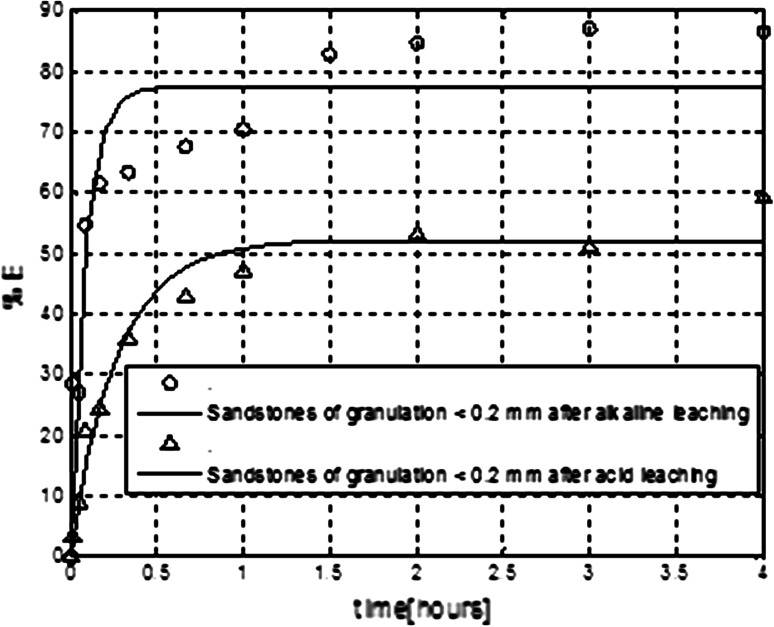
Comparison of membrane extraction of uranium performed with real solutions after acid and alkaline leaching of sandstones

After analysing the results of the experiments one can see that, the extraction efficiency of uranium was slightly higher for the samples obtained after leaching of brown shales (see Fig. 5). For this case %*E* = 79.39 and *D*_c_ = 3.85. For the real solutions obtained after leaching of sandstones %*E* = 74.22 and *D*_c_ = 2.88. For slightly higher percentage of extraction of uranium may have impact the higher initial concentrations of uranium in real solutions after leaching the sandstones. As it was demonstrated in the previous experiments with model solutions (see Fig. 4) the initial uranium concentration has fundamental importance for extraction efficiency. In this case initial concentration of uranium was 8.09 × 10^−3^ (μg/mL). For real solution after leaching of brown shales the initial concentration of uranium was lower −6.23 × 10^−3 ^(μg/mL). In case of experiments shown in Fig. 6 one can see, that the higher extraction efficiency of uranium was obtained for real solution after alkaline leaching. In this case, the initial concentration of uranium was equal 473 × 10^−3 ^(μg/mL) for real solutions after acid leaching and equal 181 × 10^−3^ (μg/mL) after alkaline leaching. The differences in extraction efficiencies for recovery of uranium after acid leaching of sandstones presented in Figs. 5 and 6 can be also attributed to different initial uranium concentrations in post-leaching solutions.

#### Extraction/re-extraction

After analysing the results of the experiments for model solutions the process of extraction/re-extraction with real solutions was conducted. The experiment of extraction/re-extraction with real solutions was run in the same way like extraction/re-extraction process for the model solutions (see the chapter “experiments with model solutions—extraction/re-extraction”). The results of the experiment are shown in Table 3.

**Table 3:** The results of the experiment for extraction/re-extraction of metals from real solution obtained after acid leaching of sandstones

Element	*C*_a_ (μg/mL)	*C*_b_ (μg/mL)	*D*	%*E*	*C*_c_ (μg/mL)	%reE	%*R*
Stripping phase—Na_2_CO_3_							
U	20.037	19.677	68.93	98.20	19.239	97.77	96.02
Th	0.134	0.111	4.90	83.05	0.109	98.20	81.34
Cu	1.992	0	0	0	0	0	0
Co	3.480	0	0.01	0.99	0	0	0
Mn	781.731	11.896	0.02	1.87	2.630	22.11	0.34
Zn	0	0	0	0	0	0	0
Cr	0	0	0	0	0	0	0
La	0.606	1.971 × 10^−2^	0.04	3.50	0.702 × 10^−2^	35.62	1.16
V	5.408	6.804 × 10^−2^	0.02	1.49	6.803 × 10^−2^	~100	1.26
Yb	0.102	8.569 × 10^−2^	5.41	84.39	6.866 × 10^−2^	80.13	67.31
Mo	0	0	0	0	0	0	0
Ni	1.050	0	0	0	0	0	0
Sb	0	0	0	0	0	0	0
Fe	543.36	317.131	1.40	58.31	51.439	16.22	2.98

For this process, apart from uranium, the other elements were also examined. In the initial solution, the highest concentration was determined for manganese and iron (see Table 3—*C*_a_). The degree of the extraction for the iron had significantly higher value than for the manganese but the degree of the re-extraction had similar value for both elements. The highest extraction, re-extraction and recovery were obtained for uranium, thorium and ytterbium. These last two elements had very small concentration in the initial solution. Among all the elements (see Table 3) the highest value of the re-extraction was obtained for the vanadium.

Concluding the results of experiments, one can notice that using extraction/re-extraction process it is possible to remove some metallic components from post-leaching liquors like Cu, Co and Ni. Such metals like Zn, Cr, Mo and Sb present in the ores were removed at the acid leaching stage. Further purification and separation of uranium from thorium, vanadium, manganese and lanthanides can be led by the sequence of ion exchange/extraction treatments.

The integrated process of extraction and re-extraction conducted in continuous mode is now under investigation. This process includes two membrane modules, one for extraction and the other for back extraction (stripping). In such a system, there is no saturation of the metal-extractant, because the reagent is continuously regenerated in the module for the re-extraction. The advantage of the integrated membrane process over one-stage installation with one single membrane module may also rely on the fact that, the overall mass transfer coefficient resulting from the integrated process can be greater than the coefficient of mass transfer obtained in a single membrane module [12].

## Conclusions

Membrane contactors such as Celgard X50-215 Microporous Hollow Fiber Membrane enable a variety of applications for recovery and/or removal of heavy metals from different process streams. They can be used for removal or recovery of metals from liquid wastes from industry and the separation of metallic contaminants from various kinds of wastewater [13, 14]. They are also used in the extraction of radionuclides from the water streams [15]. In the present work membrane contactors have been applied to recover uranium from aqueous solutions at different steps of processing of uranium ores. The resulting high levels of extraction using D2EHPA and various advantages of the membrane extraction enable it to be considered, as an alternative method for the extraction carried out in the mixer-settler arrangement. After preliminary re-extraction experiments, which have been carried out with the model solutions, high percentage of re-extraction and recovery of uranium were obtained. Experiments carried out with the real solutions after leaching uranium ores confirmed the results of preliminary, model experiments. Appropriate selection of hydrodynamic conditions in the membrane contactor eliminated the possibility of wetting the membrane and allowed stable working conditions of the apparatus. The results of experiments showed that alkaline leaching is more selective for uranium in presence of other metals but concentration of uranium is less compared to concentration of uranium after acid leaching, which is more effective. It was proved that in case of extraction/re-extraction process for real post-leaching solutions the high value of re-extraction and recovery of uranium were obtained.

## Variables

%*R*: Percent of recovery of uranium in extraction/re-extraction process
%reE: Percent of uranium re-extraction
*A* and *B*: The coefficients of the linear relationship
*C*_a_: Concentration of uranium in the feed phase (g/L), (μg/mL)
*C*_b_: Concentration of uranium in the organic phase (g/L), (μg/mL)
*C*_c_: Concentration of uranium in the stripping phase (g/L), (μg/mL)
*C*_o_: Concentration of the component in the organic phase (g/L)
*C*_w_: Concentration of the component in the water phase (g/L)
*d*: The outer diameter of the empty space in the module (m)
*D*: The diameter of the membrane module (m)
*d*_h1_: Hydraulic diameter of part of the module outside the capillaries (m)
*d*_h2_: The hydraulic diameter of the part of the module inside the capillaries (m)
*d*_i_: The inner diameter of the capillary (m)
*d*_o_: The outer diameter of the capillary (m)
*g*: Gravitational acceleration (m/s^2^)
*D*_c_: Distribution coefficient
*L*: Length of the pipe (m)
*n*: Number of capillaries
*p*_1_: Pressure at the inlet of the module (Bar)
*p*_2_: Pressure at the outlet of the module (Bar)
Re: Reynolds number
*T*: Approximation coefficient
*u*: Linear flow rate (m/s)
*V*_o_: Volume of organic phase
*V*_w_: Volume of aqueous phase
*W* and *T*: Approximation coefficient
*z*_1_ and *z*_2_: Level of the inlet and outlet (m)
*Z*_1,2_: Resistance to fluid flow along the module

## Greek symbols

*α*_1_ and *α*_2_: Coriolis coefficients
λ: Dimensionless drag coefficient
*μ*: Dynamic viscosity (kg/m* s)
*ν*: Kinematic viscosity (m^2^/s)
*ρ*: Density (kg/m^3^)

## Subscripts

1: Inlet of the module
2: Outlet of the module
a: Concentration of uranium in the feed phase
b: Concentration of uranium in the organic phase
c: Concentration of uranium in the stripping phase
h1: The hydraulic diameter outside of the capillaries
h2: The hydraulic diameter inside of the capillaries
i: The inner diameter of the capillary
o: Organic phase
w: Water phase
